# Intraoperative Acute Cardiac Tamponade as a Result of Intracardiac Perforation Requiring Emergency Continuous Pericardiocentesis and Open Sternotomy: A Case Report and Literature Review of a Rare but Fatal Complication

**DOI:** 10.7759/cureus.54701

**Published:** 2024-02-22

**Authors:** Christopher M Russo, Mitchell E Harrison, Nathaniel M Harry, John R Benjamin, Christian Popa

**Affiliations:** 1 Anesthesiology, Walter Reed National Military Medical Center, Bethesda, USA; 2 Anesthesia and Critical Care, Walter Reed National Military Medical Center, Bethesda, USA

**Keywords:** anesthesiology, interventional cardiology, pericardiocentesis, percutaneous, sternotomy, papvr, cardiac tamponade, intracardiac perforation

## Abstract

Intraoperative acute cardiac tamponade associated with iatrogenic intracardiac perforation from percutaneous interventional cardiac procedures is a rare but potentially catastrophic complication. We report a case of intraoperative acute hemopericardium caused by a left atrial (LA) perforation resulting in cardiac tamponade in a patient undergoing a baffling procedure for the correction of two anomalous pulmonary veins draining into her superior vena cava (SVC) that required continuous pericardiocentesis with autologous blood transfusion via the femoral vein and an emergency intraoperative transfer from the interventional cardiology cath lab to the cardiac operating room for an open sternotomy and primary repair. An 86-year-old female with known right-ventricular (RV) failure with preserved ejection fraction (left ventricular ejection fraction (LVEF): 50-55% on transesophageal echocardiography (TEE) one week prior) and atrial fibrillation was admitted for her third heat failure exacerbation in two months despite being adherent to her aggressive diuresis medication regimen. Upon her readmission and due to her symptomatic and seemingly refractory heart failure, the patient underwent a cardiac computer tomography (CT) with 3D reconstruction that showed previously undiagnosed partial anomalous pulmonary venous return (PAPVR) of two of her four pulmonary veins aberrantly draining into the SVC. This anatomic pathology was deemed to be the likely etiology of her repeated episodes of recurring heart failure exacerbations, shortness of breath, peripheral edema, and fatigue. The patient was counseled and consented to a percutaneous baffle of the two anomalous veins to redirect more of the returning pulmonary venous blood away from the SVC and to the LA. While under general endotracheal anesthesia (GETA) with a TEE in place during the procedure, the patient suddenly developed acute hypotension, tachycardia, and a reduction in expired carbon dioxide (EtCO_2_) was noted quickly followed by evidence of a rapidly accumulating hemopericardium on TEE. Cardiothoracic surgery was urgently consulted to the interventional cardiology cath lab while the patient underwent an emergency pericardiocentesis that momentarily alleviated her hemodynamic instability, cardiac tamponade physiology, and deteriorating overall clinical picture. While performing continuous pericardiocentesis with autologous return of the aspirated blood via femoral venous access the patient was urgently transported to the cardiac operating room and prepped for emergency sternotomy for primary repair of the LA. Following primary repair via sternotomy, multiple drains were placed and the thoracic cavity was closed with wires. The patient was immediately transported to the surgical intensive care unit (SICU) intubated, mechanically ventilated, and sedated. During this time, the patient progressively required additional vasoactive and inotropic agents to support her mean arterial pressure (MAP), and following a multidisciplinary discussion with the patient’s family regarding her goals of care, the decision was made to withdraw further resuscitation efforts and the patient expired four hours later.

## Introduction

Initially introduced in the 1960s, percutaneous cardiac interventions have exploded in growth and utility, primarily driven by diagnostic cardiac catheterization and percutaneous coronary interventions (PCI), the latter of which was originally introduced by Gruentzig in 1978 [1]. This revolutionary technical and procedural advancement is a true achievement and its list of potential applications as it relates to non-ischemic cardiac pathology continues to grow, as technology and techniques continue to improve [2]. Despite a promising record with ample data exhibiting improved overall outcomes for patients, PCI continues to be limited by significantly lower success rates, higher required revision rates, and higher complication rates in patients with abnormal anatomy and congenital anatomical variations [3]. Partial anomalous pulmonary venous return (PAPVR) is a congenital heart defect that is typically identified and corrected in the pediatric population; however, the incidence of PAPVR in the adult population is largely unknown but estimated to be roughly between 0.5% and 0.7% of the population [4,5]. PAPVR refers to conditions in which there is some degree of aberrant pulmonary venous drainage to any location other than the LA. The clinical presentation, constellation of symptoms, and overall severity of PAPVR are dependent on the amount of involved pulmonary veins and the degree of anomalous venous return. The spectrum of disease burden with PAPVR is vast and can range from completely asymptomatic to severe cardiopulmonary complications such as hypogenetic lung syndrome (Scimitar syndrome) resulting in global hypoplasia of a lung (typically the right lung) due to complete aberrant venous drainage from all involved pulmonary lobes [6]. As the burden of symptoms varies greatly with each patient’s anatomy, with PAPVR, the various treatment options range from symptomatic management to complicated and complex open cardiothoracic and vascular surgery with the most notable being the Warden procedure [7]. First described in 1984, the Warden procedure has been used for surgically correcting PAPVR via transection and reanastomosis of the SVC to the right atrial appendage and baffling of the anomalous pulmonary veins but is associated with a number of significant postoperative complications such as SVC syndrome, SVC obstruction, pulmonary venous obstruction, and acute sinus node dysfunction [8,9]. As with all invasive surgical procedures, less invasive percutaneous approaches for PAVPR have been explored and developed and now are frequently approached via intra-arterial baffling with the goal of redirecting the aberrant pulmonary venous flow toward the LA. We report a case of a catastrophic outcome of intraoperative LA perforation resulting in acute hemopericardium, cardiac tamponade requiring emergent and continuous pericardiocentesis and emergency sternotomy for primary repair.

## Case presentation

**Figure 1:**
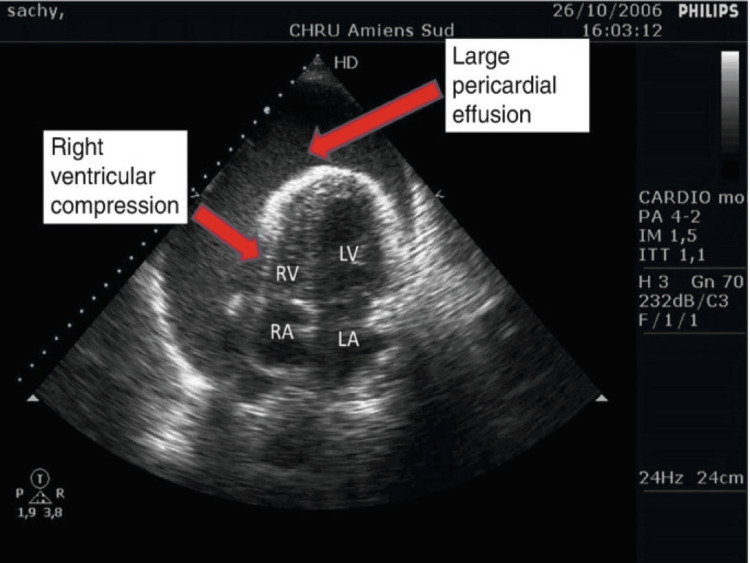
Example of large pericardial effusion and RV compression on TEE via a deep transgastric five-chamber view RV: right ventricular; TEE: transesophageal echocardiography

**Figure 2:**
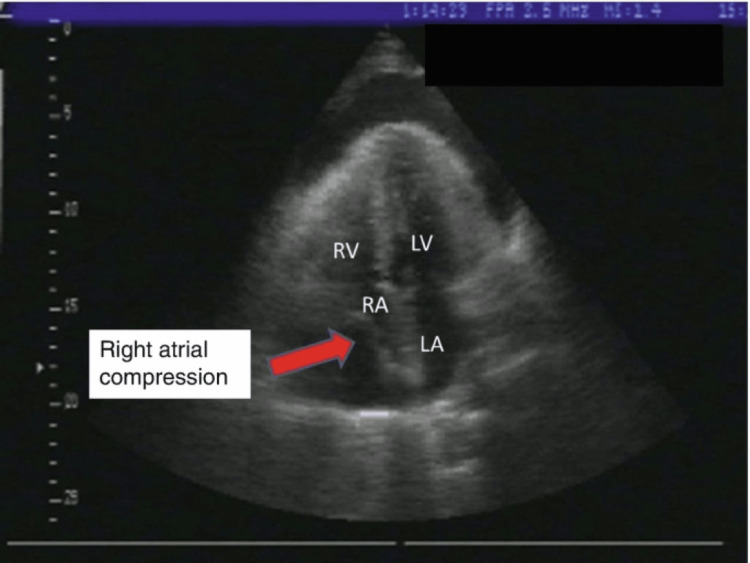
Example of RA compression on TEE via mid-esophageal four-chamber view RA: right atrial; TEE: transesophageal echocardiography

An 86-year-old female with known RV failure and preserved ejection fraction (left ventricular ejection fraction (LVEF): 50-55% on transesophageal echocardiography (TEE) one week prior), atrial fibrillation, and hypertension, who presented with shortness of breath, peripheral edema, and fatigue, was admitted inpatient for what was suspected to be her third heart failure exacerbation in the last two months despite being adherent to her aggressive diuresis medication regimen. Due to the rapid return of heart failure symptoms, the decision was made to obtain a cardiac 3D CT with reconstruction to evaluate any potential unknown underlying pathology that may be contributing to her symptoms. The 3D CT showed the patient having two of four of her pulmonary veins anomalous draining into the SVC. Interventional cardiology was consulted, as the patient underwent diuresis and was optimized for possible interventional procedures to address her pulmonary vein anomaly. Following a multidisciplinary meeting involving interventional cardiology, cardiothoracic surgery, and cardiothoracic anesthesiology, the decision was made based on her comorbidities, unstable hemodynamic profile, and significantly elevated morbidity and mortality if approached in an open surgical approach to proceed with elective baffle implantation via a minimally invasive percutaneous approach in an effort to redirect the anomalous pulmonary vein draining to the LA and, as a result, improve her congestive heart failure (CHF) and overall quality of life. The patient was stabilized and transported to the interventional cardiology cath lab the following morning. As the patient was adequately pre-oxygenated with 100% fraction of inspired oxygen (FiO2) via a mask, standard American Society of Anesthesiologist (ASA) monitors were applied, and a right-sided radial arterial line was placed under ultrasound guidance prior to the induction of anesthesia. Due to the patient’s medical comorbidities, recent aggressive diuresis, and tenuous hemodynamics, the induction of anesthesia was performed with Etomidate 0.2 mg/kg, Fentanyl 50 mcg, and Rocuronium 0.6 mg/kg without any complications. Video laryngoscopy was performed with a McGrath 3 blade, and the patient was successfully intubated with a 7.0 endotracheal tube (ETT), and end-tidal CO2 capnography was hooked up to the anesthesia circuit. The patient was then turned over to the interventional cardiology team. As the team prepared for the percutaneous approach a separate cardiologist placed a TEE probe without complication. During the intraarterial wire introduction of the percutaneous procedure, the patient became acutely tachycardic and hypotensive and showed a subsequent acute reduction in expired carbon dioxide (EtCO2) on end-tidal CO2 capnography concerning for an acute reduction in cardiac output of unknown etiology. This was initially treated with vasopressors to maintain a mean arterial pressure (MAP) above 65 mmHg. At this time, the cardiologist performing the TEE noted a rapidly evolving hemopericardium likely secondary to iatrogenic LA perforation by the intraarterial penetrating wire advancement (Figures 1, 2) [10]. The patient was stabilized with vasoactive and inotropic agents (phenylephrine and epinephrine) while the interventional cardiology called cardiothoracic surgery urgently to the cath lab. The interventional cardiology team prepped for emergency subxiphoid pericardiocentesis. Upon penetration of the pericardial sac, a large volume of blood was drawn back into a 60 cc syringe, and using a sterile technique, the blood was returned to the patient. Initially, the pericardiocentesis appeared to resolve the patient's tamponade physiology, as she was normocardic and normotensive without the need for vasopressors for a brief period of time. However, minutes later, the patient became acutely tachycardic and hypotensive, with reduced EtCO2 once again, and the TEE showed a rapid re-accumulation of blood in the pericardial sac, resulting in a return of tamponade physiology. The interventional cardiology team using multiple 60 cc syringes performed continuous pericardiocentesis while a second cardiologist returned the blood-filled syringe in an autologous fashion via femoral venous access. At this time, it was evident that until the LA underwent primary surgical repair, the patient would continue to have persistent pericardiocentesis-dependent hemopericardium and tamponade physiology. An intraoperative multidisciplinary decision was made to abandon the baffling procedure due to the patient's acute hemodynamic vulnerability, optimize the patient, perform an intraoperative transport to the cardiac operating room, and attempt exploratory surgery with primary repair of the LA via open sternotomy. During transport, we continued the continuous pericardiocentesis while retuning all aspirated blood to the patient via the femoral vein while providing 100% FiO2 via an Ambu bag. Upon arrival at the cardiac operating room, the patient’s hemodynamics were dependent on continuous pericardiocentesis and if paused for even a moment would quickly revert to tamponade physiology. The patient was transferred to the operating table, standard ASA monitors were applied, and her chest was prepped in an emergent fashion and the sternotomy was rapidly performed. A massive transfusion protocol was called at this time to have blood products available in the operating room. Primary repair of the LA was performed without any cannulation modifications and without the use of cardiopulmonary bypass during the emergency surgery. Prior to surgical closure, drains were placed in the pericardium, right pleural space, and the sternum was reapproximated and closed with stainless steel wires. Upon conclusion of the sternotomy, the patient had a significant requirement of vasoactive and inotropic agents (Milrinone, Epinephrine, Vasopressin, Dobutamine) and was urgently transferred to the SICU where she remained intubated, mechanically ventilated, and sedated. Overnight, the patient continued to have hypotension with increasing vasopressor requirements and evidence of significantly reduced cardiac output. Following a discussion with the family, care was withdrawn the following morning and the patient expired four hours postop.

## Discussion

**Figure 3:**
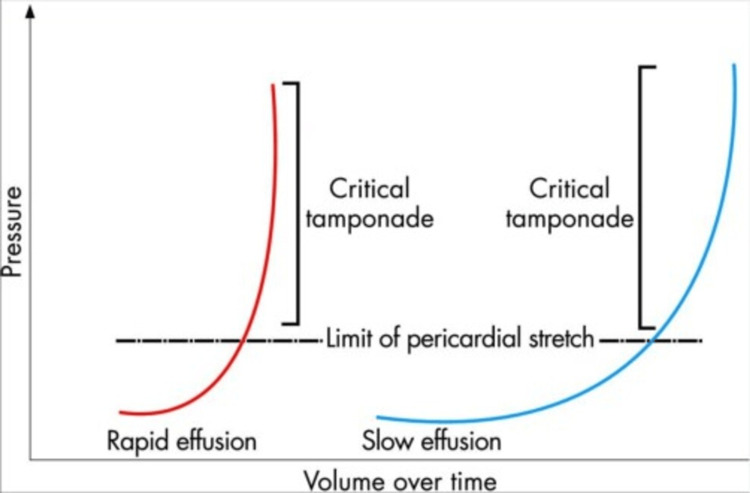
Comparison of differing slopes and points of critical tamponade of acute and chronic pericardial effusions in pressure over time

Anesthetic and surgical management of intraoperative hemopericardium and subsequent cardiac tamponade is a dynamic and challenging situation for any physician to face and requires rapid detection, attention to detail, and swift action. The pathophysiology of acute cardiac tamponade is a direct result of the pressure-volume relationship of the pericardial sac as it relates to the intraventricular pressure of the RV, as it is a significantly lower pressure system than compared to the LV. In acute cardiac tamponade, the critical reserve volume of the pericardium is rapidly exceeded typically in the setting of a previously healthy pericardium as opposed to a pericardium exposed longitudinally to chronically accumulating effusions over time. Chronic pericardial effusions, however, by nature, produce very gradual volumes with subsequent minor increases in pericardial pressure. In the setting of chronic effusion states, the pericardial sac becomes altered resulting in more hypercompliant pericardial sac tissues that function as a buffer to prevent the pressure within the pericardial sac from reaching or exceeding hemodynamically significant critical tamponade physiology (Figure 3) [11].

Due to the naturally small reserve volume capacity the pericardial sac can accommodate, even small amounts of rapid fluid accumulation can produce acute hemodynamically catastrophic changes, particularly in the setting of surgery in patients with multiple medical comorbidities. Once the pressure within the pericardial sac (irrespective of etiology or timing) reaches the critical stage and exceeds the intraventricular pressure of the RV tamponade physiology and subsequent symptomology (tachycardia, hypotension, dyspnea, elevated jugular venous pressures, reduced cardiac output) will rapidly present [12]. If the acute reduction in RV filling volume is not detected and corrected expeditiously, this will rapidly cascade into reduced cardiac preload and reduced cardiac output and can rapidly result in LV failure and ultimately subsequent complete biventricular cardiovascular collapse [13]. Diagnosis of intraoperative cardiac tamponade in a patient under general anesthesia requires close attention and strict monitoring of the patient’s hemodynamic changes and the utility of intraoperative TEE. Intraoperative TEE is the gold standard and diagnostic test of choice for rapidly detecting the presence of a developing or existing pericardial effusion in the intraoperative setting. If a physician is not credentialed or trained in TEE, subxiphoid surface TTE is an excellent noninvasive rapid diagnostic modality. The most sensitive TEE findings for pericardial tamponade are the presence of fluid within the pericardial sac and evidence of RA collapse while the most specific findings are LA collapse and paradoxical motion of the interventricular septum, commonly referred to as septal displacement or “septal bowing” [14]. An electrocardiogram (ECG) can also aid in establishing a diagnosis of cardiac tamponade classically presenting as tachycardia with diffuse low voltage and electrical alternans due to the anterior-posterior swinging of the heart within the fluid-filled pericardial sac [15]. Following rapid identification, the treatment of pericardial tamponade involves immediate hemodynamic and inotropic support with emergency pericardiocentesis, and if refractory, a pericardial window, and lastly, the only definitive treatment for permanent pericardial constriction, a surgical pericardiectomy [16].

## Conclusions

This patient with a complicated symptomatic cardiac history of RV failure, atrial fibrillation, and a congenital defect in PAPVR underwent planned percutaneous baffling of her two anomalous veins aberrantly draining into her SVC. This case was not only medically challenging in the preoperative phase due to her medical comorbidities, challenging surgical optimization and induction of anesthesia, but also intraoperatively, where she suffered a complex and catastrophic complication of intracardiac perforation of the LA resulting in acute hemopericardium and cardiac tamponade that required emergent and continuous pericardiocentesis and intraoperative transfer to the cardiac OR for emergency sternotomy for primary repair. This case serves as a reminder of the risk of percutaneous/transcatheter interventional cardiac procedures for the anesthesiologist, interventional cardiologist, and cardiothoracic surgeon while also highlighting the importance of the rapid identification and treatment of acute intraoperative cardiac tamponade.
